# Socioeconomic status and remaining teeth in Japan: results from the Toyama dementia survey

**DOI:** 10.1186/s12889-019-7068-7

**Published:** 2019-06-04

**Authors:** Nobue Nakahori, Michikazu Sekine, Masaaki Yamada, Takashi Tatsuse, Hideki Kido, Michio Suzuki

**Affiliations:** 1 Faculty of Nursing Science, Tsuruga Nursing University, 78-2-1 Kizaki, Tsuruga, Fukui 914-0814 Japan; 2 Department of Epidemiology and Health Policy, Graduate School of Medicine and Pharmaceutical Sciences, University of Toyama, 2630 Sugitani, Toyama, Toyama 930-0194 Japan; 3 Kiseikai, Kido Clinic, 244 Honoki, Imizu, Toyama 934-0053 Japan; 4 Department of Neuropsychiatry, Graduate School of Medicine and Pharmaceutical Sciences, University of Toyama, 2630 Sugitani, Toyama, Toyama 930-0194 Japan

**Keywords:** Remaining teeth, Socioeconomic status, Educational attainment, Occupation

## Abstract

**Background:**

The prevalence of periodontal disease is increasing among elderly individuals in Japan. Reports on the risk factors for tooth loss have included socioeconomic status (SES); however, few studies have addressed the association between remaining teeth and SES by examining whether education and occupation have a synergistic effect on tooth loss. Accordingly, the present study evaluated the association of remaining teeth with the socioeconomic factors of educational and occupational histories in Japanese elderly individuals.

**Methods:**

This retrospective case-control study used data from the Toyama Dementia Survey, Japan. Toyama Prefecture residents aged ≥65 years were randomly selected for the study (sampling rate, 0.5%), and 1303 residents agreed to participate (response rate, 84.8%). Structured interviews with participants and family members (or proxies, if necessary) were conducted. Participants’ lifestyle factors (e.g., smoking and alcohol consumption), medical history, and SES (educational and occupational history) as well as the presence or absence of remaining teeth were assessed. The association between SES and remaining teeth was examined using a logistic regression analysis.

**Results:**

Overall, 275 cases with no remaining teeth and 898 controls with remaining teeth were identified. The odds ratio (OR) for complete tooth loss was higher among less educated participants (≤6 years) than among highly educated participants [age- and sex-adjusted OR, 3.29; 95% confidence interval (CI), 1.90–5.71]; furthermore, it was higher among participants with a blue-collar occupational history than among those with a white-collar occupational history (age- and sex-adjusted OR, 2.16; 95% CI, 1.52–3.06). After adjusting for employment history or educational attainment, the ORs for tooth loss were 2.79–3.07 among less educated participants and 1.89–1.93 among participants with a blue-collar occupational history. A current or former smoking habit and medical history of diabetes and osteoporosis increased the risk of tooth loss. The interaction term of a low level of education and a history of blue-collar occupation with tooth loss was not significant.

**Conclusions:**

In Japan, a low SES is a risk factor for tooth loss. A low level of education is a more important predictor of tooth loss than a blue-collar occupation.

## Background

In Japan, the extension of a healthy life expectancy, healthcare, and disease prevention among elderly individuals are important issues. In humans, a decline in oral function can impact the entire body, leading to lower motor function and dementia [1]. In elderly individuals, a decline in the oral function may be attributed to conditions such as tooth loss, which is a consequence of other oral disorders, such as periodontal disease and caries. Accordingly, tooth loss has been described as a consequence of accumulation of dental diseases associated with aging.

Among elderly individuals, the proportion of those retaining ≥20 teeth at the age of 80 years has gradually increased from approximately 10% in the 1980s to approximately 50% in 2011. Although this increase in the remaining teeth among elderly individuals is encouraging, these teeth must be prevented from any harm due to periodontal disease. Over time, the percentage of individuals with a periodontal pocket with a depth of ≥4 mm has increased among individuals aged ≥65 years, despite declining trends among those aged < 64 years [2]. Furthermore, periodontal disease has been reported as the main cause of tooth loss among individuals aged ≥45 years [3]. Therefore, early prevention of periodontal disease is essential for preserving remaining teeth among elderly individuals.

In addition to the conditions related to oral hygiene (e.g., periodontal disease), lifestyle factors, such as eating habits, alcohol consumption, and smoking, as well as systemic diseases, such as diabetes, have been reported as risk factors for tooth loss [4]. Moreover, correlations of socioeconomic factors with periodontal disease and tooth loss have been reported [5–7]. However, few studies have examined how socioeconomic status (SES) affects remaining teeth among elderly individuals in Japan [8]; furthermore, few studies have analyzed the association of remaining teeth with SES using an interaction term of educational and occupational histories [9]. Therefore, this study aimed to clarify the influence of SES on remaining teeth in Japanese elderly individuals. An understanding of this association could help identify strategies for avoiding the risk of tooth loss in elderly individuals.

## Methods

The Toyama Dementia Survey was a random sample survey of elderly individuals aged ≥65 years living in the Toyama Prefecture, Japan. The survey screened elderly individuals for dementia and collected data on the use of appropriate dementia care. This cross-sectional survey was administered in 1985, 1990, 1996, 2001, and 2014. The present study was based on the results of the most recent survey, conducted in 2014 [10, 11].

### Participants

A 0.5% random sample of the 307,582 Toyama Prefecture residents aged ≥65 years on October 1, 2013, was identified using computers employing the basic resident register. Thus, 1537 people living at home and in elderly care facilities were chosen. A total of 1303 (84.8%) people agreed to participate in the study. Public health nurses telephoned the selected individuals and explained the purpose of the research. Once consent was obtained, the nurses visited the participants at a later date and interviewed them regarding the presence or absence of remaining teeth, age, sex, educational attainment level, occupational history, lifestyle factors, and medical history. Family members and institution staff supported this process as necessary and provided data when the participants were unable to respond. Finally, complete responses from 1173 participants (275 cases with no remaining teeth and 898 controls with remaining teeth) were analyzed.

Participation in the Toyama Dementia Study was voluntary. All participants or their family members provided written informed consent prior to the study. The University of Toyama ethics committee approved the study protocol.

### Remaining teeth

Participants were classified as having “with no remaining teeth” or “with remaining teeth.” Participants with no remaining teeth were defined as those who used complete dentures or had no teeth or dentures. Participants with remaining teeth were defined as those who chewed mainly with their own teeth or partial dentures.

### Demographic factors and SES

For all participants, age, sex, educational attainment level, occupational history, lifestyle factors, and medical history were recorded. The educational attainment level and occupational history were examined under SES assessment; educational attainment was stratified into the following categories: ≤6 years (elementary school), 7–9 years (higher than elementary school or through junior high school), and ≥ 10 years (high school, junior high school under the old system, girls’ high school under the old system, high school under the old system, technical school, or university). Occupations were classified as white-collar, blue-collar, both, or other in accordance with the Japan Standard Occupational Classification [12]. White-collar employment included administrative or managerial, specialized professional, clerical, sales, and service positions. Blue-collar employment was defined as positions in security, agriculture, forestry or fisheries, manufacturing, transport or machine operations, construction or mining, carrying, cleaning, packaging, and others. “Both” referred to those who had held both white- and blue-collar jobs, whereas “other” referred to those who had held other types of employment (e.g., housewifery).

### Lifestyle factors

The participants’ alcohol consumption and smoking habits were examined, and they were classified as current, former, or nondrinkers. Current drinkers comprised those who consumed alcohol daily, occasionally, or within the past year; former drinkers comprised those who had ceased consuming alcohol at least 1 year before the study; and nondrinkers comprised those who had never habitually consumed alcohol. Similarly, participants were classified as current, former, or nonsmokers. Current smokers comprised those who smoked daily or had smoked within the previous year; former smokers comprised those who had stopped smoking at least 1 year before the study; and nonsmokers comprised those who had never habitually smoked.

### Medical history

The participants’ medical histories were assessed in accordance with the 10th revision of the International Statistical Classification of Diseases and Related Health Problems [13]. The following systemic diseases were identified as conditions of interest regarding the absence of remaining teeth: diabetes, hypertension, hyperlipidemia, stroke, angina pectoris/cardiovascular disease, malignant tumor, and osteoporosis.

### Statistical analysis

Chi-square test was used to identify the differences in socioeconomic, lifestyle, or medical history variables between the participants with no and with remaining teeth. A logistic regression analysis was used to test the potential association of SES with remaining teeth; here remaining teeth was set as the dependent variable and age, sex, lifestyle factors, and medical history were set as the independent variables. We created several multivariate models to test whether the lifestyle factors or medical histories explained any observed associations between socioeconomic factors and remaining teeth [14]. First, we calculated the age- and sex-adjusted odds ratios (ORs) for remaining teeth based on educational attainment level, occupational history, and socioeconomic factors. Subsequently, lifestyle variables and then medical history variables were added to the model [14].

To further investigate the association between SES and remaining teeth, an interaction term of educational attainment level and occupational history was tested. We examined the fitness of this logistic regression model using the Hosmer–Lemeshow test. SPSS version 23 (IBM Inc., Armonk, NY, USA) was used to conduct all statistical analyses. ORs and 95% confidence intervals (CIs) were calculated based on the logistic regression analysis.

## Results

Table 1 presents the demographic characteristics of the participants. The proportion of participants with no remaining teeth differed significantly depending on age; educational attainment level; occupation; smoking; history of diabetes, stroke, angina pectoris/cardiovascular disease, and osteoporosis.

**Table 1:** Participant Characteristics

			Remaining teeth				*P*
			No (*n* = 275)		Yes (*n* = 898)		
			*n*	%	*n*	%	
Attribution	Age (years)	65–74	56	20.4	512	57.0	< 0.001
		75–84	114	41.5	308	34.3	
		≥85	105	38.2	78	8.7	
	Sex	Male	129	46.9	379	42.2	0.186
		Female	146	53.1	519	57.8	
Educational attainment	Years of education	≤6	48	17.5	30	3.3	< 0.001
		7–9	112	40.7	318	35.4	
		≥10	115	41.8	550	61.2	
Occupation	Type of occupation	White-collar	73	26.5	393	43.8	< 0.001
		Blue-collar	137	49.8	295	32.9	
		Both	42	15.3	154	17.1	
		Other	23	8.4	56	6.2	
Lifestyle	Alcohol consumption habit	Current drinker	63	22.9	329	36.6	< 0.001
		Former drinker	42	15.3	65	7.2	
		Nondrinker	170	61.8	504	56.1	
	Smoking habit	Current smoker	33	12.0	79	8.8	0.016
		Former smoker	87	31.6	228	25.4	
		Nonsmoker	155	56.4	591	65.8	
Medical History	Hypertension	Yes	137	49.8	410	45.7	0.240
		No	138	50.2	488	54.3	
	Hyperlipidemia	Yes	41	14.9	166	18.5	0.205
		No	234	85.1	732	81.5	
	Diabetes	Yes	59	21.5	131	14.6	0.009
		No	216	78.5	767	85.4	
	Stroke	Yes	31	11.3	70	7.8	0.085
		No	244	88.7	828	92.2	
	Angina pectoris/ cardiovascular disease	Yes	37	13.5	69	7.7	0.005
		No	238	86.5	829	92.3	
	Malignant tumor	Yes	31	11.3	101	11.2	1.000
		No	244	88.7	797	88.8	
	Osteoporosis	Yes	40	14.5	82	9.1	0.013
		No	235	85.5	816	90.9	

Table 2 presents the effects of educational attainment level and occupation on the incidence of no remaining teeth based on an unadjusted analysis and an analysis adjusted for lifestyle variables and medical history. The proportion of individuals with no remaining teeth increased as SES decreased. Specifically, participants with an educational attainment level of ≤6 or 7–9 years were less likely than those with the level of ≥10 years to have remaining teeth, with age- and sex-adjusted ORs of 3.29 (95% CI: 1.90–5.71; model 1) and 1.57 (95% CI: 1.14–2.16; model 1), respectively. After adjusting the models for occupation, lifestyle factors, and medical history, the ORs for an educational attainment level of ≤6 years increased to 2.79–3.07 (models 3–5), whereas the ORs for the level of 7–9 years did not differ significantly from those for the level of ≥10 years.

**Table 2:** Association of remaining teeth with socioeconomic status after adjusting for lifestyle factors and medical history

	Crude odds	Model; odds ratio (95% confidence interval)				
		1	2	3	4	5
Educational attainment						
≤ 6 years	7.65 (4.65–12.60)	3.29 (1.90–5.71)		2.84 (1.62–4.99)	2.79 (1.58–4.93)	3.07 (1.73–5.45)
7–9 years	1.68 (1.26–2.26)	1.57 (1.14–2.16)		1.32 (0.94–1.85)	1.33 (0.94–1.87)	1.38 (0.97–1.95)
≥ 10 years	1.00	1.00		1.00	1.00	1.00
Occupation						
White-collar	1.00		1.00	1.00	1.00	1.00
Blue-collar	2.50 (1.81–3.45)		2.16 (1.52–3.06)	1.89 (1.31–2.73)	1.91 (1.32–2.79)	1.93 (1.32–2.82)
Both	1.47 (0.96–2.24)		1.50 (0.95–2.37)	1.38 (0.87–2.21)	1.39 (0.86–2.23)	1.34 (0.83–2.17)
Other	2.21 (1.28–3.82)		1.76 (0.96–3.22)	1.71 (0.93–3.15)	1.69 (0.91–3.14)	1.71 (0.91–3.20)
Age						
65–74 years	1.00	1.00	1.00	1.00	1.00	1.00
75–84 years	3.38 (2.39–4.80)	3.12 (2.18–4.46)	3.30 (2.31–4.71)	3.03 (2.12–4.35)	3.05 (2.10–4.41)	2.89 (1.98–4.22)
≥ 85 years	12.31 (8.23–18.41)	10.73 (6.96–16.55)	13.04 (8.57–19.83)	10.48 (6.77–16.23)	10.26 (6.53–16.12)	9.85 (6.21–15.63)
Sex						
Male	1.21 (0.92–1.59)	1.82 (1.33–2.49)	1.60 (1.17–2.19)	1.75 (1.27–2.41)	1.34 (0.77–2.32)	1.43 (0.82–2.51)
Female	1.00	1.00	1.00	1.00	1.00	1.00
Alcohol consumption						
Current drinker	0.57 (0.41–0.78)				0.43 (0.28–0.67)	0.44 (0.28–0.68)
Former drinker	1.92 (1.25–2.93)				1.19 (0.71–2.00)	1.18 (0.70–2.00)
Nondrinker	1.00				1.00	1.00
Smoking						
Current smoker	1.59 (1.02–2.48)				3.79 (1.98–7.27)	4.05 (2.11–7.78)
Former smoker	1.46 (1.07–1.97)				1.95 (1.14–3.34)	2.00 (1.16–3.42)
Nonsmoker	1.00				1.00	1.00
Hypertension						
Yes	1.18 (0.90–1.55)					1.15 (0.84–1.58)
No	1.00					1.00
Hyperlipidemia						
Yes	0.77 (0.53–1.12)					0.85 (0.56–1.31)
No	1.00					1.00
Diabetes						
Yes	1.60 (1.14–2.25)					1.72 (1.16–2.55)
No	1.00					1.00
Stroke						
Yes	1.50 (0.96–2.35)					1.26 (0.74–2.12)
No	1.00					1.00
Angina pectoris/cardiovascular disease						
Yes	1.87 (1.22–2.86)					1.10 (0.68–1.80)
No	1.00					1.00
Malignant tumor						
Yes	1.00 (0.65–1.54)					0.81 (0.50–1.33)
No	1.00					1.00
Osteoporosis						
Yes	1.69 (1.13–2.54)					1.88 (1.16–3.05)
No	1.00					1.00

Model 1 was adjusted for age, sex, and educational attainment

Model 2 was adjusted for age, sex, and occupation

Model 3 was adjusted for age, sex, and socioeconomic status

Model 4 was adjusted for age, sex, lifestyle, and socioeconomic status

Model 5 was adjusted for age, sex, lifestyle, medical history, and socioeconomic status

Blue-collar workers were also less likely than white-collar workers to have remaining teeth, with an age- and sex-adjusted OR of 2.16 (95% CI 1.52–3.06; model 2). After adjusting the models for educational attainment level, lifestyle factors, and medical history as well, the ORs for a blue-collar job history increased to 1.89–1.93 (models 3–5). After adjusting for all potential confounders (model 5), a low educational attainment level, older age, current or former smoking habit, history of diabetes, and history of osteoporosis were found to be the factors that increase the risk of tooth loss. In contrast, current alcohol consumption was not found to increase this risk.

After adjusting for the interaction term of educational attainment level and occupational history in model 5, ORs of low educational attainment level and a blue-collar occupational history for remaining teeth hardly changed (3.08 and 1.79, respectively). Thus, the interaction term was not significant.

## Discussion

The present study found that a low SES, as indicated by a low educational attainment level (≤6 years) and blue-collar occupational history, was a risk factor for complete tooth loss in elderly individuals. The interaction term of a low educational attainment level and a blue-collar occupational history with tooth loss was not significant.

We also observed that an older age significantly increased the risk of complete tooth loss, which is attributable to the accumulation of dental diseases with age; in other words, elderly individuals are more likely to lose their teeth.

We further observed that a current and former smoking status were strong risk factors for tooth loss, consistent with the results of previous studies [15, 16]. Tobacco smoke contains approximately 5300 chemicals, of which at least 250 are harmful. These chemicals enter the blood circulation and can cause cancer, arteriosclerosis, tissue inflammation, and other disorders. Notably, the oral cavity is the organ first exposed to tobacco smoke. Smoking enhances the pathogenicity of oral cavity bacteria and simultaneously impairs immune responses and wound healing, leading to periodontal disease and caries. Furthermore, the chemicals contained in tobacco also suppress gingival bleeding and harden the gingiva. This can conceal the symptoms of periodontal disease and simultaneously reduce the peripheral blood flow, reducing the capacity for healing in the oral cavity. Thus, smoking is directly related to tooth loss. The Health Promotion Law prevents the risk of passive smoking exposure [17]. However, prior to the enactment of this law in 2002, smoking was more frequent and common in the workplace. This background may have led to the oral health deterioration observed in participants in this study. Conversely, this study found that a current drinking habit reduced the risk of complete tooth loss. Although previous studies have noted that excessive drinking is associated with periodontal disease [18, 19], the mechanism linking drinking to periodontal disease remains to be elucidated. Further studies of the association between alcohol consumption and tooth loss are warranted.

The finding that a significant association exists between diabetes and the absence of remaining teeth is consistent with the results of previous studies [20, 21]. The hyperglycemia associated with diabetes causes blood vascular damage and increases susceptibility to infection, including periodontal disease, which involves the entry of infectious bacteria into the gingiva and blood vessels. Furthermore, periodontal disease causes gingival inflammation, and the resulting secretion of inflammatory substances into blood circulation interferes with the ability of insulin to reduce blood glucose levels. Thus, diabetes and periodontal disease exert reciprocal negative effects. The results of the present study suggest that diabetes prevention and control can help prevent tooth loss. The study also revealed a significant association between osteoporosis and remaining teeth. Periodontal disease is likely to worsen in participants with osteoporosis because they have poor bone mass in oral cavity [22], which is shown by the results of this study. The prevalence of osteoporosis is particularly high in postmenopausal women. Thus, it is important to think about countermeasures by paying attention to osteoporosis when attempting prevention of tooth loss in people aged ≥65 years.

The present study further identified a blue-collar occupational history as a risk factor for tooth loss, consistent with a previous report that workers in skilled trades, manufacturing, sales, transportation, and communications face a higher risk of periodontal disease than those in professional and technical fields [23]. Similarly, transportation drivers have been reported to have a greater risk of tooth loss than white-collar workers [24]. Blue-collar workers tended to remain on work sites and to work in three-shift cycles, which may lead to irregular meal times and, consequently, irregular tooth brushing habits. Thus, the lifestyle related to blue-collar employment is more likely to lead to tooth loss.

Additionally, participants with an educational attainment level of ≤6 years had an approximately 2–3 fold greater risk of tooth loss than those with a level of ≥10, even after adjusting for age, sex, occupation, lifestyle, and medical history. Educational attainment level may affect lifestyle habits, as children of more highly educated parents have been reported to brush their teeth more frequently per day and to have fewer dental caries [25, 26], suggesting that highly educated families have better dental hygiene habits. The parental educational history may also influence the formation of a child’s teeth. Tooth development begins during early pregnancy and requires a balanced inoculation of protein, calcium, and various vitamins. A previous study has reported ingestion of a nutritionally balanced diet only among individuals with a high SES [27]. Therefore, a low household SES may affect the nutritional intake of the mother and the fetal tooth development, leading to less healthy teeth later in life. Furthermore, previous reports have suggested that individuals with a low educational attainment level do not tend to participate in medical checkups [28], whereas those with a low SES tend to be less able to maintain health [29]. Therefore, we speculated that individuals with a low SES are less likely to take countermeasures to address the risks of caries and periodontal disease, regardless of their access to relevant information.

Education is followed by an appropriate occupation; thus, a low educational attainment level has a higher influence on tooth loss than the occupational history [28, 30]. The oral cavity is the only internal organ that can be examined visually and treated directly. Although individuals may not notice the signs of early tooth decay and periodontal disease, dental plaque can be removed easily using a toothbrush or dental floss. Although an individual’s SES may affect the dental hygiene level and tooth strength, countermeasures that broaden one’s knowledge about oral care and enable the acquisition of good dental lifestyle habits (e.g., tooth brushing) may overcome the effects of a low SES. Furthermore, a social system that enables access to periodic dental examinations throughout a person’s lifetime and an environment supporting proper oral care are important.

However, this study has several limitations. First, the responses were collected retrospectively rather than ad hoc, which might have affected the accuracy of the data. Second, we may have selected participants who were relatively more cooperative and led relatively healthier lifestyles, which could have introduced selection bias. Third, the study design may have led to a risk of survival bias, as we were unable to collect data from individuals who died during the survey period. Fourth, we did not examine local factors, such as oral hygiene and dietary habits, and therefore, we may have overestimated the association between socioeconomic factors and tooth loss. Fifth, we did not collect the data on remaining teeth number, and it may have led to an insufficient analysis. Despite these limitations, our study meaningfully confirms that both socioeconomic factors and systemic illnesses related to tooth loss have an effect on the maintenance of remaining teeth.

## Conclusions

The present study found that a low educational attainment level (≤6 years) and a blue-collar occupational history are independently associated with remaining teeth in an elderly Japanese population. Furthermore, in this population, educational attainment level had a relatively more important influence on the risk of tooth loss compared with the occupational history.

## Data Availability

The datasets analyzed during the current study are available from the corresponding author on reasonable request.

## Abbreviations

CI: Confidence interval
OR: Odds ratio
SES: Socioeconomic status

## References

[1] Tsakos G, Watt RG, Rouxel PL, Oliveira C, Demakakos P (2015). Tooth loss associated with physical and cognitive decline in older adults. J Am Geriatr Soc.

[2] Health and Labour Statistics Association (2018). Proportion of persons with more than 20 teeth.

[3] Aida J, Ando Y, Akheter R, Aoyama H, Matsui M, Morita M (2006). Reasons for permanent tooth extractions in Japan. J Epidemiol..

[4] Amano A, Yasui T, Miyazaki H, Tsurumoto A, Kawaguchi Y, Yamashita Y, Hirose K (2017). Periodontal disease prevention. Oral Health and Preventive Dentistry.

[5] Buchwald S, Kocher T, Biffar R, Harb A, Holtfreter B, Meisel P (2013). Tooth loss and periodontitis by socio-economic status and inflammation in a longitudinal population-based study. J Clin Periodontol.

[6] Wamala S, Merlo J, Bostrom G (2006). Inequity in access to dental care services explains current socioeconomic disparities in oral health: the Swedish National Surveys of public health 2004-2005. J Epidemiol Community Health.

[7] Gilbert GH, Paul Duncan R, Shelton BJ (2003). Social determinants of tooth loss. Health Serv Res.

[8] Tashiro A, Aida J, Hobugawa Y, Shobugawa U, Fujiyama Y, Yamamoto T (2017). Association between income inequality and dental status in Japanese older adults: analysis of data from JAGES 2013. Nihon Koshu Eisei Zasshi.

[9] Yamamoto T, Kondo K, Aida J, Fuchida S, Hirata Y, Group J (2014). Association between the longest job and oral health: Japan Gerontological Evaluation Study project cross-sectional study. BMC Oral Health.

[10] Toyama Prefectural Health and Welfare. Toyama Dementia Survey in 2014. https://www.pref.toyama.jp/cms_sec/1211/kj00000125-011-01.html. Accessed 16 Feb 2019.

[11] Nakahori N, Sekine M, Yamada M, Tatsuse T, Kido H, Suzuki M (2018). A pathway from low socioeconomic status to dementia in Japan: results from the Toyama dementia survey. BMC Geriatr.

[12] Ministry of Internal Affairs and Communications. Japan Standard Occupational Classification (rev. 5th, December 2009). www.soumu.go.jp/english/dgpp_ss/seido/shokgyou/co09-4.htm. Accessed 15 Oct 2018.

[13] World Health Organization. International Statistical Classification of Diseases and Related Health Problems (ICD) 10th revision (February 2016). https://icd.who.int/browse10/2010/en. Accessed 15 Oct 2018.

[14] Hosmer DW, Lemeshow S, Sturdevant RX (2013). Applied logistic regression.

[15] Hanioka T, Ojima M, Tanaka K, Matsuo K, Sato F, Tanaka H (2011). Causal assessment of smoking and tooth loss: a systematic review of observational studies. BMC Public Health.

[16] Albandar JM, Streckfus CF, Adesanya MR, Winn DM (2000). Cigar, pipe, and cigaratte smoking as risk factors for periodontal disease and tooth loss. J Periodontol.

[17] Cabinet Office. Health Promotion Law. https://www8.cao.go.jp/kisei-kaikaku/oto/otodb/english/houseido/hou/lh_9999-69.html. Accessed 27 Apr 2019.

[18] Tezal M, Grossi SG, Ho AW, Genco RJ (2004). Alcohol consumption and periodontal disease. J Clin Periodontol.

[19] Pitiphat W, Merchant AT, Rimm EB, Joshipura KJ (2003). Alcohol consumption increases periodontitis risk. J Dent Res.

[20] Kaur G, Holtfreter B, Rathmann WG, Schwahn C, Wallaschofski H, Schipf S (2009). Association between type 1 and type 2 diabetes with periodontal disease and tooth loss. J Clin Periodontol.

[21] Ohtake T, Takahashi R, Ohyabu Y, Minamisono N, Kuzuyama T, Ohishi K (2005). A study on community periodontal index of treatment needs (CPITN) in type 2 diabetic patients. Nihon Shishubyo Gakkai Kaishi.

[22] Yoshihara A, Seida Y, Hanada N, Miyazaki H (2004). A longitudinal study of the relationship between periodontal disease and bone mineral density in community-dwelling older adults. J Clin Periodontol.

[23] Irie K, Yamazaki T, Yoshii S, Takeyama H, Shimazaki Y (2017). Is there an occupational status gradient in the development of periodontal disease in Japanese workers? A 5-year prospective cohort study. J Epidemiol.

[24] Suzuki S, Yoshino K, Takayanagi A, Ishizuka Y, Satou R, Kamijo H (2016). Comparison of risk factors for tooth loss between professional drivers and white-collar workers: an internet survey. Ind Health.

[25] Kato H, Tanaka K, Shimizu K, Nagata C, Furukawa S, Arakawa M (2017). Parental occupations, educational levels, and income and prevalence of dental caries in 3-year-old Japanese children. Environ Health Prev Med.

[26] Schou L, Uitenbroek D (1995). Social and behavioral indicators of caries experience in 5-year-old children. Community Dent Oral Epidemiol.

[27] Darmon N, Drewnowski A (2008). Does social class predict diet quality?. Am J Clin Nutr.

[28] Broadbent JM, Thomson WM, Boyens JV, Poulton R (2011). Dental plaque and oral health during the first 32 years of life. J Am Dent Assoc.

[29] Togari T, Yamazaki Y (2009). The socioeconomic gradient of sense of coherence-from a representative sample survey of 4800 Japanese people aged 20 to 40. Bull Soc Med.

[30] Poulton R, Caspi A, Milne BJ, Thomson WM, Taylor A, Sears MR (2002). Association between children's experience of socioeconomic disadvantage and adult health: a life-course study. Lancet.

